# Interplay between thyroid cancer cells and macrophages: effects on IL-32 mediated cell death and thyroid cancer cell migration

**DOI:** 10.1007/s13402-019-00457-9

**Published:** 2019-06-14

**Authors:** Yvette J. E. Sloot, Katrin Rabold, Thomas Ulas, Dennis M. De Graaf, Bas Heinhuis, Kristian Händler, Joachim L. Schultze, Mihai G. Netea, Johannes W. A. Smit, Leo A. B. Joosten, Romana T. Netea-Maier

**Affiliations:** 1 Department of Internal Medicine (463), Division of Endocrinology, Radboud University Medical Center, Geert Grooteplein Zuid 8, 6525 GA Nijmegen, The Netherlands; 2 Radiotherapy & OncoImmunology Laboratory, Department of Radiation Oncology, Radboud University Medical Center, Geert Grooteplein 32, 6525 GA Nijmegen, The Netherlands; 3 Genomics & Immunoregulation, LIMES-Institute, University of Bonn, Bonn, Germany; 4 Department of Medicine, University of Colorado Denver, Aurora, CO 80045 USA; 5 Department of Internal Medicine and Radboud Center for Infectious Diseases (RCI), Radboud University Medical Centre, Nijmegen, Geert Grooteplein Zuid 8, 6525 GA Nijmegen, The Netherlands; 6 PRECISE Platform for Single Cell Genomics and Epigenomics, German Center for Neurodegenerative Diseases and University of Bonn, Bonn, Germany; 7 Department of Medical Genetics, Iuliu Hatieganu University of Medicine and Pharmacy, 400349 Cluj-Napoca, Romania

**Keywords:** Thyroid cancer, IL-32, Cancer cell migration, Cancer cell death

## Abstract

**Purpose:**

Interleukin 32 (IL-32) is a pro-inflammatory cytokine of which different isoforms have been identified. Recently, IL-32 has been shown to act as a potent inducer of cell migration in several types of cancer. Although previous research showed that IL-32 is expressed in differentiated thyroid cancer (TC) cells, the role of IL-32 in TC cell migration has not been investigated. Furthermore, tumour-associated macrophages (TAMs) may play a facilitating role in cancer cell migration. The aim of this study was to explore whether the interaction between TC cells and TAMs results in increased expression of IL-32 in TC cells and to investigate whether this affects TC cell migration.

**Methods:**

TPC-1 cells were co-culture with TC-induced or naive macrophages. Next, transcriptome analysis on TPC-1 cells was performed and supernatants were used for stimulation of TPC-1 cells. IL-32β and IL-32γ were exogenously overexpressed in TPC-1 cells using transient transfection, after which an in vitro gap closure assay was performed to assess cell migration, and the expression of migratory factors was assessed using RT-qPCR.

**Results:**

We found that TC-induced macrophages induced IL-32 expression in TC cells and that TAM-derived TNFα was the main inducer of IL-32β expression in TC cells. Overexpression of IL-32β and IL-32γ did not affect TC cell migration, but increased cell death. Finally, we found that IL-32β overexpression led to increased mRNA expression of the pro-survival cytokine IL-8, while the expression of other migratory factors was not affected.

**Conclusions:**

From our data, we conclude that TAM-derived TNFα induces IL-32β in TC cells. Although IL-32β does not affect TC cell migration, alternative splicing of IL-32 towards the IL-32β isoform may be beneficial for TC cell survival through induction of the pro-survival cytokine IL-8.

**Electronic supplementary material:**

The online version of this article (10.1007/s13402-019-00457-9) contains supplementary material, which is available to authorized users.

## Introduction

Interleukin 32 (IL-32) is a pro-inflammatory cytokine that has first been described in 2005 [1]. Since its discovery, nine different isoforms of IL-32 have been identified, all resulting from alternative splicing of IL-32γ pre-mRNA [2, 3]. IL-32 has been linked to the pathogenesis of several diseases, including inflammatory diseases, cardiovascular diseases and cancer [3–9]. Especially in the context of cancer, the role of IL-32 seems ambiguous. Several reports point towards a tumour suppressive role for IL-32 [9–12], with IL-32β and IL-32γ being associated with inhibition of melanoma and colon cancer cell growth [10] and with induction of an antitumour immune response in prostate and colon cancer cells [12]. In contrast, other reports indicated that IL-32 may have a more pro-tumorigenic function, especially with respect to mediating invasion and migration in lung, breast and gastric cancers and in osteosarcoma, effects that have not been linked to a specific isoform [13–16]. Thus, IL-32 seems to exert opposite effects in the context of different cell- and cancer types, which also depends on which isoform is present [9].

The role of IL-32 in non-medullary, differentiated thyroid cancer (TC) has only scarcely been investigated. Previously, we have shown that IL-32 protein is expressed in TC, and mRNA expression analyses showed that both the IL-32β and IL-32γ isoforms were present in TC tissues [17]. Furthermore, a single nucleotide polymorphism (SNP) in the promoter of IL-32, resulting in increased IL-32γ production in immune cells, has been found to be associated with an increased risk of developing TC [17] and an increased resistance to conventional therapy. Moreover, it has been found that IL-32β and IL-32γ expression can lead to the induction of caspase-8 dependent cell death in two different TC cell lines (FTC-133, follicular PTEN-deficient TC; and BCPAP, papillary BRAF V600E mutated TC) [18].

Previous research suggests that IL-32 may act as a potent inducer of cell migration in other cancers [13–16], but the effect of IL-32 expression on TC cell migration and metastasis has not been investigated. The process of metastasis is complex and involves several components of the tumour microenvironment (TME), including pluripotent tumour-associated macrophages (TAMs), which may play a facilitating role. The TME of poorly differentiated TCs is highly infiltrated with TAMs and this infiltration has been found to be correlated with a poor prognosis [19, 20]. The mechanisms through which IL-32 is produced in TCs are not known, and knowledge of the effects of IL-32 on pro-carcinogenic processes is lacking. TAMs secrete high amounts of cytokines such as TNFα in response to interactions with TC cells [21], and TNFα has been shown to be one of the main inducers of IL-32 [1, 3, 4]. We thus hypothesized that IL-32 expression in TC cells may increase in response to the presence of activated immune cells, i.e., TAMs, in the TME. This may, in turn, result in an increased IL-32 mediated migration of the tumour cells, potentially contributing to the metastatic process. Therefore, the aim of the present study was to explore whether the interaction between TC cells and innate immune cells induces increased expression of IL-32 in TC cells, and to investigate whether this affects TC cell migration and/or death in differentiated TCs.

To test this hypothesis, we first assessed the effect of the interaction between TAMs and TC cells in an in vitro co-culture model of human monocytes and a TC-derived cell line (TPC-1). To further assess the effect of IL-32 on TC cell migration, TC cells were transfected with IL-32β and IL-32γ after which cell migration in an in vitro gap closure assay and mRNA expression of migratory factors known to be associated with IL-32 expression were analysed. These included IL-8, matrix metalloproteinases (MMPs), the epithelial-to-mesenchymal transition (EMT) marker E-cadherin and vascular endothelial growth factor (VEGF) [13–16].

## Materials and methods

### Co-culture model of the TC cell line TPC-1 and human monocytes

The co-culture in vitro experiments were performed using TPC-1 cells (papillary, RET/ PTC rearrangement) [22]. TPC-1 cells were grown in RPMI-1640 culture medium, Dutch modification (Life Technologies, Carlsbad, CA, USA) supplemented with gentamycin 50 μg/ml, pyruvate 1 mM, GlutamMAX 2 mM and 10% Fetal Calf Serum (FCS, Gibco, Life Technologies). Peripheral blood mononuclear cells (PBMCs) were isolated by density gradient centrifugation using Ficoll-plaque (GE Healthcare, Diegem Belgium) from buffy coats obtained from Sanquin Bloodbank, Nijmegen, The Netherlands. Ethical approval was provided by CMO Arnhem-Nijmegen (CMO 2010–104) and all experiments were performed in accordance with the principles expressed in the Declaration of Helsinki. For transcriptome analysis, an additional step of purification using CD14-labelled magnetic beads was performed after PBMC isolation in order to obtain a highly purified monocyte population.

A trans-well system with ThinCert™ cell culture inserts in a 24-well plate (Greiner Bio-One GmbH, Austria) was used for co-culture of TC cells and human monocytes. Cell counts were performed using a Coulter particle counter (Beckman Coulter Inc. Pasadena, CA, USA). A total of 1.0 × 10^5^ TPC-1 cells in 250 μl culture medium was added to the upper compartments of the trans-well system. Upper compartments solely with medium were used as negative controls. Culture medium was added to the lower compartments and the cells were incubated for 24 h at 37 °C, 5% CO_2_. Next, the cells were washed with phosphate buffered saline (PBS, Braun Melsungen, Germany) after which fresh co-culture medium, containing a physiological concentration of glucose, was added: RPMI-1640 without glucose (Life Technologies, Carlsbad, California, USA) supplemented with glucose 5 mM, pyruvate 1 mM, gentamicin 50 μg/ml and HEPES 10 mM (Life Technologies, Carlsbad, California, USA).

Monocytes were isolated as described above and resuspended in the co-culture medium. A total of 5.0 × 10^5^ monocytes in 500 μl was added to the lower compartments of the trans-well system. After adhesion for 1 h in the 24-well plates, the non-adherent cells were discarded, and the adherent monocytes were incubated further with TPC-1 cells or medium alone for 24 h at 37 °C in co-culture medium. After 24 h incubation, the adherent monocytes, now called naïve monocytes or TC-induced macrophages, respectively, were stimulated for 24 h with RPMI-1640 or 10 ng/ml LPS (*E. coli* strain O55:B5, Sigma Chemical Co, St. Louis, MO, USA) as substitute for endogenous TLR4 ligand signalling, while leaving the trans-wells containing medium or TPC-1 cells in place. At the end of the incubation period, TPC-1 cells were lysed in 200 μl Trizol reagent (Invitrogen, Carlsbad, CA, USA) and stored at −80 °C for transcriptome analysis. Supernatants where collected and stored at −80 °C for subsequent conditioned medium experiments. A schematic representation of the experimental set-up is provided in Supplementary Fig. S1.

### RNA sequencing of TPC-1 cells co-cultured with monocytes

RNA isolation was performed according to the Trizol manufacturer’s instructions (Invitrogen, Carlsbad, CA, USA). 50 ng RNA was converted into cDNA libraries according to the TruSeq RNA library preparation kit v2. After cluster generation on a cBot (Illumina), a 75 bp single read (SR) rapid run was performed on an Illumina HiSeq 1500 system.

### RNA-Seq pre-processing and statistical analysis of samples

After base calling and de-multiplexing using CASAVA version 1.8, the 75 bp single-end reads were aligned to the human reference transcriptome hg38 from UCSC by kallisto v0.44.0 using default parameters. Data were imported into DESeq2 (v.1.10.1) using the TXimport (v1.2.0) package. DESeq2 was used for the calculation of normalized counts for each transcript using default parameters. After DESeq2 normalization, all normalized transcripts with a maximum over all group means lower than 10 were excluded resulting in 17,099 present genes. Unwanted or hidden sources of variation, such as batch and preparation date, were removed using the sva package [23]. The normalized rlog transformed expression values were adjusted according to the two surrogate variables identified by sva using the function removeBatchEffect from the limma package [24]. Differentially expressed (DE) genes were defined by a |fold change| > 1.5 and an adjusted *p* value (FDR) < 0.05. Log_2_ fold changes were plotted against –log_10_ adjusted *p *values in a volcano plot. Top 10 up- and down-regulated genes were marked in the volcano plot. Sashimi plots were generated by visualizing the aligned reads using the Integrative Genome Viewer (IGV) [25], and the function sashimi plot. Principal component analysis was performed on all present genes. Detailed data have been deposited in the GEO database with accession number GSE120391.

### Stimulation of TC cell lines with conditioned medium and TNFα

To test whether induction of IL-32 mRNA expression in TPC-1 was caused by paracrine factors from the co-culture medium, fresh TPC-1 cells were stimulated with supernatants from TPC-1-monocyte co-culture experiments for 24 h. Since TNFα is an important inducer of IL-32 [1, 4] and TNFα is abundantly present in the co-culture medium [21], TPC-1 cells were also stimulated with TNFα (100 ng/ml R&D Systems, Minneapolis, MN, USA) for 24 h. Briefly, 500.000 cells/well were seeded in a 24-well plate (Corning, New York, USA) and left to adhere for 18 H. Medium was replaced with fresh medium mixed with supernatants from co-culture experiments in a 1:1 ratio or fresh medium with TNFα (100 ng/ml). To test whether IL-32 could also be induced in other TC cell lines, two additional cell lines were included, i.e., BC-PAP (papillary TC, BRAF V600E mutation) and FTC-133 (follicular TC, PTEN deficient) [22]. To confirm the role of TNFα, specific TNFα inhibitors, etanercept (decoy receptor, 10 μg/ml, Enbrel©, Pfizer, New York, USA) and adalimumab (monoclonal antibody, 10 μg/m, Humira©, Abbott GmbH & Co. Wiesbaden, Germany) were added to pre-selected experiments.

### Expression vectors and transient transfection of TPC-1 cells

pcDNA3-based vectors for the expression of IL-32β and IL-32γ_mutant_ were generated by standard PCR and restriction based cloning methods. The expression plasmids were constructed with a Kozak sequence (5′-GCCGCCACC-3′) immediately upstream of the ATG start codon followed by full-length cDNAs of human IL-32β, IL-32γ and eGFP or an empty DNA (control). An IL-32γ_mutant_ was created that cannot be spliced into other IL-32 isoforms like regular IL-32γ, to investigate the effect of IL-32γ without the effect of over-expression of other IL-32 splice variants. The IL-32γ_mutant_ was created by mutation of a donor splice site from GU to AU as described previously [2].

TPC-1 cells were transfected with pCDNA3 plasmids expressing IL-32β, IL-32γ_mutant_ or empty DNA, together with pCDNA3 plasmids expressing eGFP in a 10:1 ratio. Briefly, cells were seeded at a density of approximately 40.000 cells per cm^2^ in a 10 mm dish (VWR, Radnor, PA, USA). Medium was refreshed after 24 h and cells were transfected using Fugene (Promega Corporation, Madison, WI, USA) according to the manufacturers’ protocol. After 24 h, cells were collected using trypsin/EDTA (Life Technologies), counted using a Bürker-Türk counter chamber (Sigma Aldrich Chemie, Zwijndrecht, The Netherlands) and Tryphan blue staining (Sigma), and only live cells were reseeded for further experiments.

### Transfection efficiency and Ki-67 staining

To quantify the transfection efficiency, 200.000 cells were reseeded after transfection. After 40 h and 64 h, the cells were trypsinised, centrifugated and fixed through resuspension in Fix and Perm buffer (Life technologies) for 45 min. After washing in Perm buffer, the cells were stained with an anti-Ki-67 monoclonal antibody (SolA15, invitrogen, Carlsbad, CA, USA) as a marker for proliferation activity, and incubated for 30 min in the dark at 4 °C. Next, the cells were washed and resuspended in PBS containing 1% Bovine Serum Albumin (BSA, Sigma-Aldrich Chemie, Zwijndrecht, The Netherlands) for analysis by flow cytometry. A Cytoflex flow cytometer (3 lasers, Beckman Coulter Inc.) was used to determine the percentage of transfected cells (i.e., GFP expressing cells) by setting the excitation at 488 nm, the emission at 525 nm (FITC channel) and recording 100.000 events per sample. The percentage of Ki-67 positive cells was determined by setting the excitation at 488 nm and the emission at 610 nm (ECD-A channel). The percentage of positive cells was obtained using a fluorescence intensity histogram, which represents the number of events at a given fluorescence intensity.

### Cytotoxicity assay

To assess cytotoxicity, we performed a Cytotox96 assay (Promega, Madison, WI, USA) according to the manufacturer’s protocol.

### Western blotting

To assess IL-32 protein expression, cells were seeded at a density of 300.000 cells/well in a 24-well plate (Corning). At two different time points, 40 and 64 h after transfection, cells were lysed in lysis buffer containing phosphatase and protease inhibitors (Roche, Basel, Switzerland) after which proteins were loaded on a pre-casted 4–15% gel (Biorad, CA, USA) for polyacrylamide gel electrophoresis. The separated proteins were transferred to a nitrocellulose membrane (Biorad) after which incubation overnight at room temperature with a goat-anti-IL-32 (AF3040) antibody (R&D systems, Minneapolis, Minnesota, USA) was used to detect IL-32 protein. Detection of actin with a rabbit anti-actin antibody (Sigma) was used to verify protein concentrations. Polyclonal secondary antibodies (Dako, Begium) and a Clarity Western ECL Blotting Substrate (Biorad) were used to visualize protein expression.

### RNA isolation, cDNA synthesis and RT-qPCR

For mRNA expression analyses, TPC-1 cells (200.000/well) were lysed in TRIzol reagent (Invitrogen, Carlsbad, CA, USA) and stored at −80 °C. RNA isolation was performed according to the manufacturer’s instructions and transcribed into cDNA by reverse transcription using an iScript cDNA Synthesis Kit (Bio-Rad). A Power SYBR Green PCR Master Mix (Applied Biosystems, CA, USA) was used for RT-qPCR in a CFX384 Touch Real-Time PCR Detection System (Bio-Rad). Expression data were normalized to the housekeeping genes human β_2_M or GAPDH. The primers used were purchased from Biolegio (Nijmegen, The Netherlands) and the sequences used for RT-qPCR are listed in Supplementary Table S1. In the different stimulation experiments the mRNA expression levels of IL-32 isoforms α, β and γ were assessed. After transfection, the mRNA expression levels of migratory factors associated with IL-32 expression were assessed, including IL-8, MMP2, 3 and 9, the EMT-marker E-cadherin and VEGF [13–16]. The mRNA expression level of IL-32α was also assessed, as this is an IL-32 splicing product and expression of IL‐32β and IL‐32γ could, therefore, also result in an increased IL‐32α mRNA level.

### Gap closure assay

In vitro gap closure assays were conducted using silicone cell culture inserts (Ibidi GmbH, Martinsried Germany) attached to culture plates. TPC-1 cells were seeded into the inserts (28.000 cells/insert chamber) and left to adhere overnight to form a confluent monolayer. Next, the inserts were removed with tweezers, and the cells were rinsed with PBS once to remove detached cells. Normal RPMI-1640 culture medium containing either 0% or 10% FCS was added after which gap closure was assessed at different time points using conventional light microscopy. Gap closure was quantified by ImageJ, using the MRI wound healing tool (National Institute of Health, Bethesda, MD, USA).

### Statistical analysis

All analyses were performed in Graphpad prism 5 (CA, USA). Differences in mRNA expression levels, GFP and Ki-67 positivity, cytotoxicity and differences in gap closure, were analysed using Kraskal Wallis test with Dunnets multiple comparison test, or Mann Whitney test to compare different conditions to control condition, or paired Wilcoxon signed rank test to compare effects within conditions. * *p* < 0.05, ** *p* < 0.01. Data are shown as means ± SEM.

## Results

### Co-culture of TPC-1 cells and monocytes leads to increased IL-32α and IL-32β mRNA expression in TC cells

**Fig. 1, F, first panel: IL-32α:**
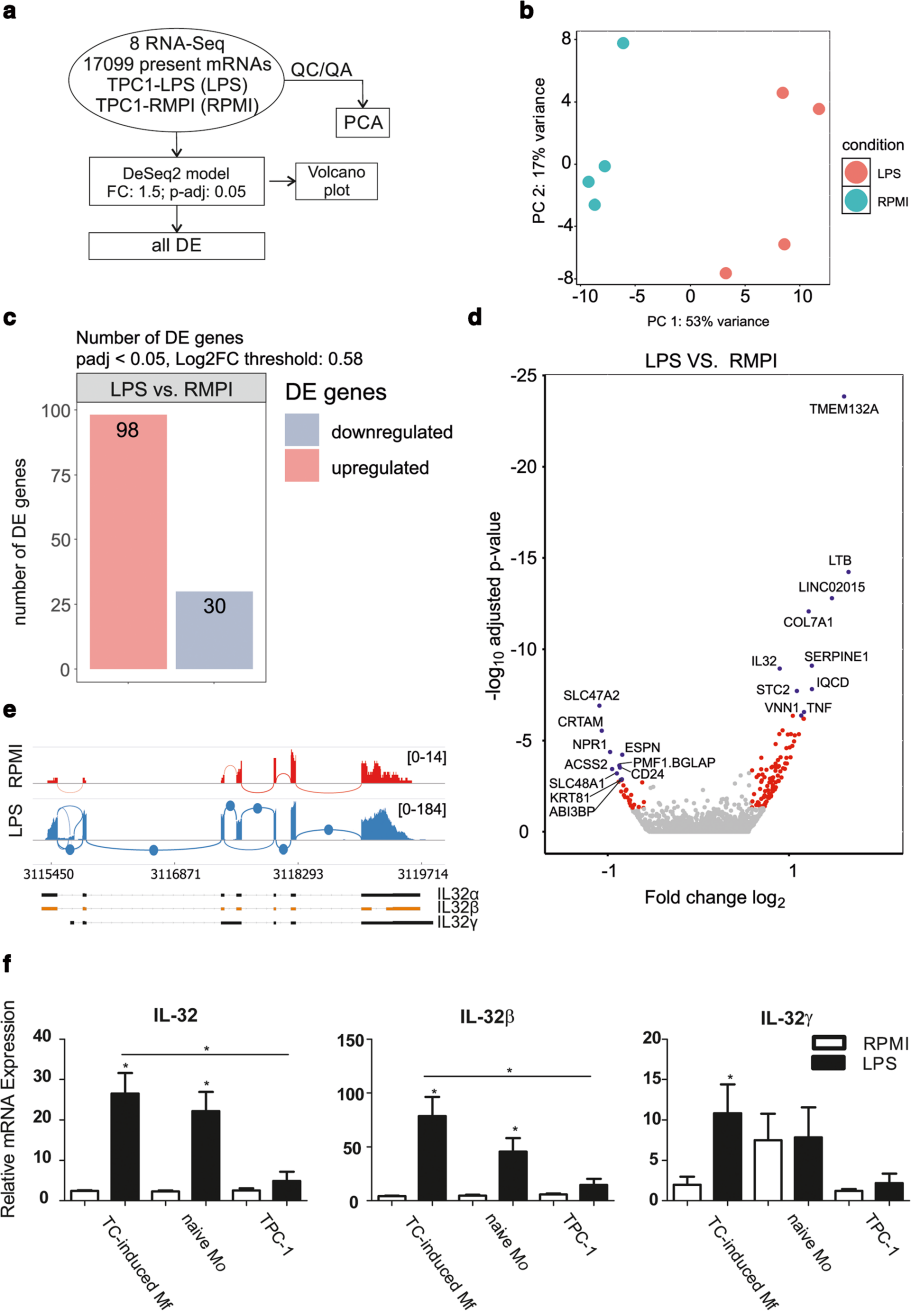
**Co-culture of TPC-1 cells with TC-induced monocytes induces IL-32 expression.** (**a**) Bioinformatics data analysis workflow of transcriptomic changes induced in TPC-1 cells co-cultured with TC-induced monocytes (4 monocyte donors) and re-stimulation with RPMI-1640 (medium control) or LPS (TLR-4 ligand). QC/QA = quality control/quality assurance. (**b**) PCA of the transcriptome data, showing the group relationships of TPC-1 cells co-cultured with TC-induced monocytes after re-stimulation with RPMI-1640 (RPMI) or LPS (LPS). The proportion of component variance is indicated as a percentage. (**c**) Bar graph showing the number of differentially expressed transcripts with fold change of 1.5 (log2 fold change of 0.58) and adjusted *p* value < 0.05. (**d**) Volcano plot of the transcriptional changes of TPC-1 cells co-cultured with TC-induced monocytes after re-stimulation with RPMI-1640 (RPMI) or LPS (LPS). The X-axis specifies the log_2_ fold-changes (FC) and the Y-axis specifies the *p*-values as the negative logarithm to the base 10 of the t-test. Red and blue dots represent transcripts expressed at significantly higher (*n* = 98) or lower (*n* = 30) levels after LPS re-stimulation. (**e**) Sashimi plots quantitatively visualizing the splice junctions for TPC-1 cells co-cultured with TC-induced monocytes after re-stimulation with RPMI-1640 (RPMI) or LPS (LPS) based on their alignment to the reference genome. (**f**) Stimulation of TPC-1 cells with supernatants from TC-induced monocytes (*n* = 8), naive monocytes (*n* = 8) and TPC-1 cells alone (*n* = 4) after re-stimulation with RPMI-1640 (medium control) or LPS (TLR-4 ligand). Results from 4 experiments, using 2 separate monocyte-donors per experiment. Data are represented as mean ± SEM; * *p* < 0.05, by Wilcoxon matched-pairs signed rank test

To investigate transcriptional differences of TPC-1 cells after co-culture with inactivated (control, RPMI-1640 stimulated) or TLR-4 activated (LPS stimulated) TC-induced-macrophages on a global level, we generated genome-wide transcriptome data through quantitative RNA-sequencing in TPC-1 cells and performed bioinformatic analyses (Fig. 1a). Unbiased principle component analysis (PCA) based on 17,099 transcripts (Fig. 1b) revealed significant transcriptional changes in TPC-1 cells after co-culture with TLR-4 activated TC-induced macrophages. Differential gene expression analysis (FC ≥ 1.5) revealed 98 significantly upregulated and 30 significantly downregulated genes (Fig. 1c). Indeed, *IL-32* was among the top 10 upregulated genes as visualized by Volcano Plot (Fig. 1d). Sashimi plots (Fig. 1e) show the splice junctions for TPC-1 cells co-cultured with TC-induced macrophages after re-stimulation with RPMI-1640 (control) or TLR-4 ligand (LPS), indicating that IL-32β is the most probable splice variant.

Next, we found that stimulation of TPC-1 cells with supernatants from TC-induced macrophages, naive monocytes and TPC-1 cells only, confirmed that paracrine factors from activated TC-induced macrophages induced IL-32 expression in TPC-1 cells (Fig. 1f). We also found that supernatants from TLR-4 stimulated TC-induced macrophages induced significantly higher IL-32α, IL-32β and IL-32γ mRNA expression levels in TPC-1 cells (*p = 0.0156*) compared to supernatants from RPMI stimulated TC-induced macrophages. Supernatants from TLR-4 stimulated TC-induced macrophages were found to induce in the highest levels of IL-32β, with a fold change of 16.77 ± 6.17 compared to 9.23 ± 5.55 in naive, TLR-4 stimulated monocytes (*p = 0.0313*).

### TNFα is a strong inducer of IL-32β in TC cells

**Fig. 2:**
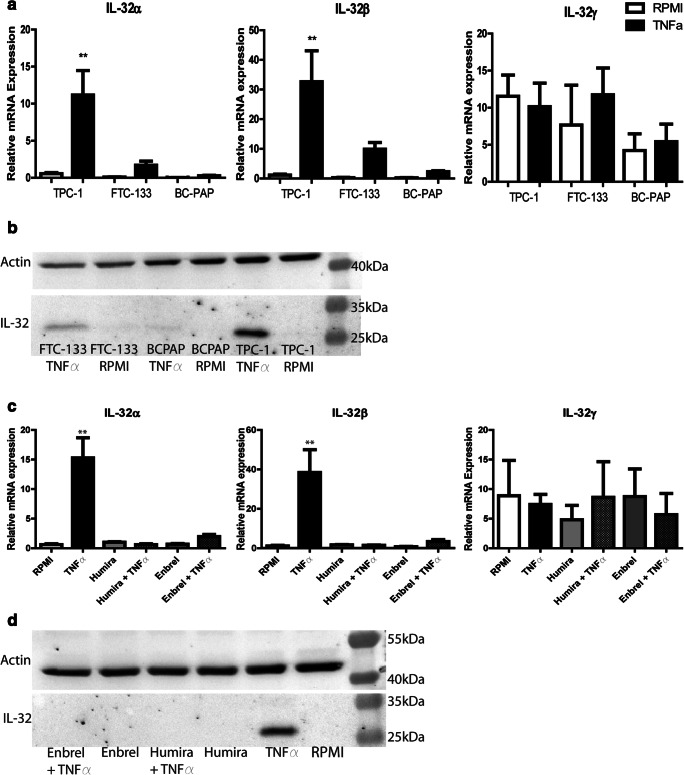
**TNFα-mediated IL-32 mRNA and protein expression in different TC cell lines.** (**a**) Relative mRNA expression of IL-32α, IL-32β and IL-32γ isoforms in 3 different TC cell lines, TPC-1 (RET/PTC rearrangement), FTC-133 (PTEN-deficient) and BC-PAP (BRAF V600E mutation) after stimulation with RPMI-1640 (medium control) or TNFα (100 ng/ml). Results from 3 to 5 experiments, including 1–3 replicates. (**b**) Western blot analysis showing IL-32 protein expression after stimulation with TNFα in TPC-1, FTC-133 and BC-PAP cells. (**c**) Relative mRNA expression levels of IL-32α, IL-32β and IL-32γ isoforms in TPC-1 cells after stimulation with TNFα and in the presence of Enbrel (etanercept, decoy TNFα-receptor, 10 μg/ml) or Humira (adalimumab, monoclonal TNFα antibody, 10 μg/ml). Results from 3 to 5 experiments, including 1–3 replicates. (**d**) Western blot analysis showing IL-32 protein expression in TPC-1 cells after stimulation with TNFα and in the presence of Enbrel or Humira. Data are presented as mean ± SEM; * *p* < 0.05, ** *p* < 0.01, *** *p* < 0.001 by Mann-Whitney-U test or Kruskal Wallis test with Dunn’s multiple comparison test

We have previously shown that TC-induced macrophages produce increased levels of TNFα and IL-6 compared to naive monocytes [21]. Since TNFα is one of the main inducers of IL-32, we hypothesized that the high levels of TNFα in the co-culture supernatants might be responsible for the rise in IL-32 mRNA expression in TC. To test this hypothesis, TPC-1 cells were stimulated with TNFα (100 ng/ml), after which again increased mRNA expression levels of IL-32α (*p = 0.0079*) and IL-32β (*p = 0.0079*) were observed, whereas the IL-32γ expression level was not significantly altered (Fig. 2a). To investigate whether TNFα could also induce IL-32 in other TC-derived cell lines, BC-PAP (papillary TC, BRAF V600E mutation) and FTC-133 (follicular TC, PTEN deficient) cells were stimulated with TNFα as well. In both BC-PAP and FTC-133 cells the IL-32α and IL-32β mRNA expression levels were found to be upregulated after stimulation with TNFα, albeit to a lesser extent (Fig. 2a).

Using Western-blot analysis, we confirmed that TNFα induced IL-32 expression at the protein level in all three TC-derived cell lines (Fig. 2b). The highest level was found in TPC-1 cells, most likely being IL-32β as based on protein size (25.9 kDa, Supplementary Fig. S2A). We found that the use of two different inhibitors of TNFα, Humira and Enbrel, abrogated the upregulation of IL-32 in the TC cell lines, further confirming that TNFα acts as an important inducer of IL-32β mRNA and protein expression in TC (Fig. 2c, d and Supplementary Fig. S2B-D).

### IL-32 overexpression results in increased cytotoxicity without affecting Ki-67 in TC cells

**Fig. 3:**
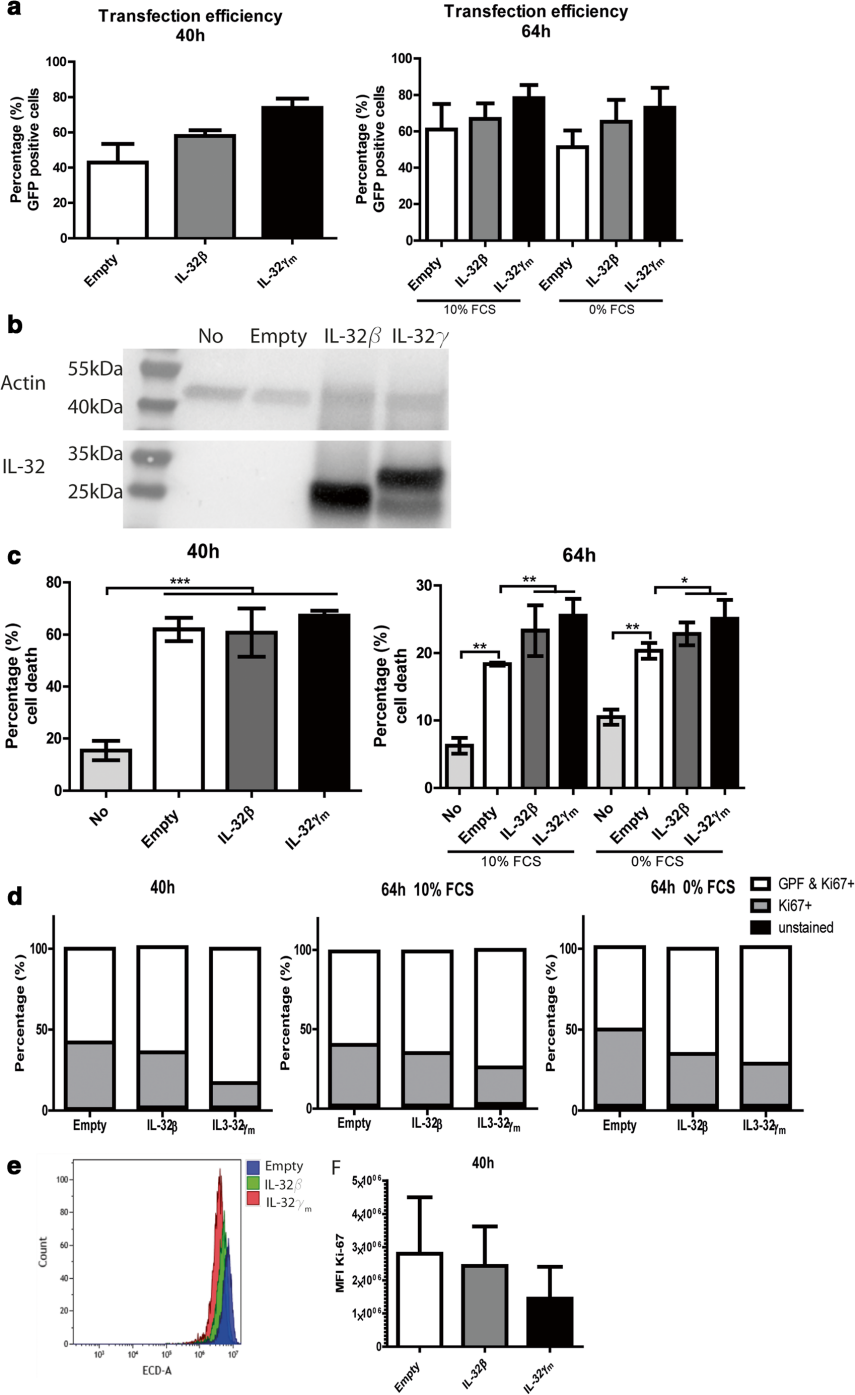
**Overexpression of IL-32β and IL-32γ in TPC-1 cells.** (**a**) Transfection efficiency determined by GPF positivity using flow cytometry at 40 h and 64 h post-transfection. Results from 3 experiments. (**b**) Western blot analysis showing overexpression of IL-32β and IL-32γ protein in TPC-1 cells 40 h after transfection, while control transfected cells (empty) show no IL-32 protein expression. (**c**) Cytotoxicity measured at 40 h (10% FCS, *n* = 4) and 64 h post-transfection (0% and 10% FCS, *n* = 3) in non-transfected cells, control transfected cells (empty) and IL-32β and IL-32γ_mutant_ transfected cells. Results from 3 to 4 experiments, 1–3 replicates per experiment. (**d**) Expression of Ki-67 proliferation marker in transfected TPC-1 cells using flow cytometry. White portion of the bar indicates GFP and Ki67 positive cells, grey portion of the bar indicates Ki67-only positive cells and black portion of the bar indicates unstained cells. (**e**-**f**) Mean fluorescence Intensity (MFI) of Ki-67 staining 40 h after transfection. Results from 3 experiments. Data are presented as mean ± SEM; * *p* < 0.05, ** *p* < 0.01, *** *p* < 0.001 by Mann-Whitney-U test or Kruskal Wallis test with Dunn’s multiple comparison test

Since IL-32 mRNA and protein expression was highest in TPC-1 cells, we next exogenously overexpressed IL-32β and IL-32γ in TPC-1 cells. IL-32β was used because this isoform was most highly expressed in TC. Since IL-32γ is known as most potent isoform of IL-32 for several functions [3], IL-32γ was also included in these experiments. To investigate the effect of IL-32γ without the effect of overexpression of other IL-32 splice variants, an IL-32γ_mutant_ vector was created, which cannot be spliced into other IL-32 isoforms like regular IL-32γ. The transfection efficacy was assessed by calculating the percentage of green fluorescent protein (GFP)-positive cells at two different time points [t = 40 h (start of gap closure assay), and t = 64 h, (end of gap closure assay)]. We found that the mean percentage of GFP-positive cells at 40 h after transfection in serum-rich conditions (10% FCS) did not differ significantly, and was lowest in the control transfected cells (46.2 ± 14.1%) and highest in the IL-32γ_mutant_ transfected cells (74.0 ± 9.2%) (Fig. 3a). Sixty-four hours after transfection, the mean percentage of GFP-positive cells was only slightly increased (not significant) in both serum-rich (10% FCS) and serum-deprived (0% FCS) conditions. In addition, we found that transfection with either IL-32β or IL-32γ_mutant_ resulted in high levels of protein expression of the indicated IL-32 isoform in TPC-1 cells at both time points (Fig. 3b, t = 40 h; data for t = 64 h not shown), while control transfected cells (empty vector) showed no IL-32 protein expression. Interestingly, we detected both IL-32β and IL-32γ protein in the 32γ_mutant_ transfected cells.

Next, the effect of exogenous IL-32 overexpression on cytotoxicity was assessed. An increased cytotoxicity was observed at 40 h after transfection for all conditions compared to non-transfected cells (*p = 0.0006*) (Fig. 3c), indicating that transfection did result in increased cell death. At 64 h post transfection, we found lower percentages of cytotoxicity due to transfection, and that both IL-32β and IL-32γ overexpression induced significantly more cytotoxicity both in the presence of serum (*p = 0.0043* and *p = 0.0022)* and in the absence of serum (*p = 0.0411* and *p = 0.0129*). IL-32γ overexpression resulted in the highest level of cell death in the presence of serum (25.5 ± 3.3% cytotoxicity for IL-32γ transfected cells compared to 18.4 ± 0.5% in control transfected cells).

To investigate the effect of IL-32 on proliferation, we analyzed whether the expression of Ki-67, a marker for proliferation, was affected by exogenous IL‐32 overexpression. We found that > 98% of all cells were Ki‐67 positive in all conditions, and that all GFP-positive cells were also Ki-67 positive (Fig. 3d). The mean fluorescence intensity of Ki-67 staining in IL-32γ_mutant_ transfected cells was found to be lower compared to that in control transfected cells and IL-32β transfected cells, but the differences were not statistically significant (Fig. 3e, f). Thus, we conclude that IL-32 overexpression did not affect the percentage of Ki-67-positive cells and only slightly affected Ki-67 intensity.

### IL-32 overexpression does not affect TC cell migration

**Fig. 4:**
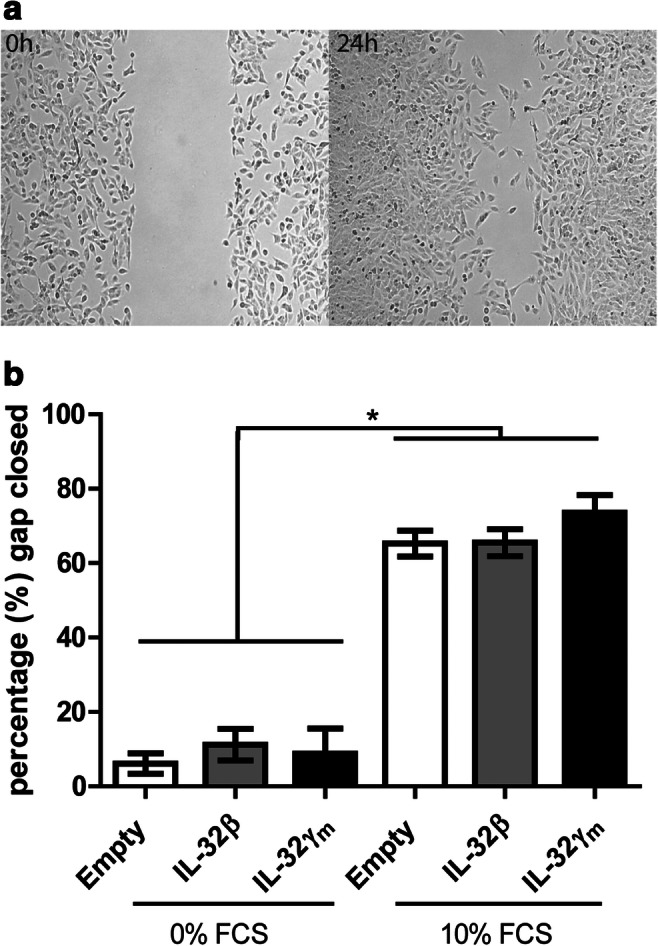
**Overexpression of IL-32β and IL-32γ in TPC-1 cells does not affect gap closure.** (**a**) The effect of IL-32β and IL-32γ overexpression on TC cell migration was assessed in a gap closure assay using IBIDI© inserts to create a cell-free zone. Gap closure was assessed after 24 h using the MRI wound healing tool in ImageJ. (**b**) The effect of IL-32β (*n* = 5) and IL-32γ (*n* = 3) overexpression on TC cell migration in serum-deprived (0% FCS) or serum-rich conditions (10% FCS) depicted as percentage of the gap that is closed after 24 h. Results from 3 to 5 experiments, 2–3 replicates per experiment. Data are represented as mean ± SEM; * *p* < 0.05, by Mann-Whitney-U test

Next, the effect of exogenous IL-32β and IL-32γ overexpression on TC cell migration in an in vitro gap closure assay was assessed (see example in Fig. 4a). Figure 4b shows the percentage of the gap that the cells filled in a 24 h period. As expected, we found the highest percentage of gap closure in serum-rich conditions with an abundance of growth and migratory factors. When comparing control transfected cells with IL-32β or IL-32γ transfected cells, no significant differences were found in the percentages of gap closure after 24 h, neither in serum-deprived nor in serum-rich conditions.

**Fig. 5:**
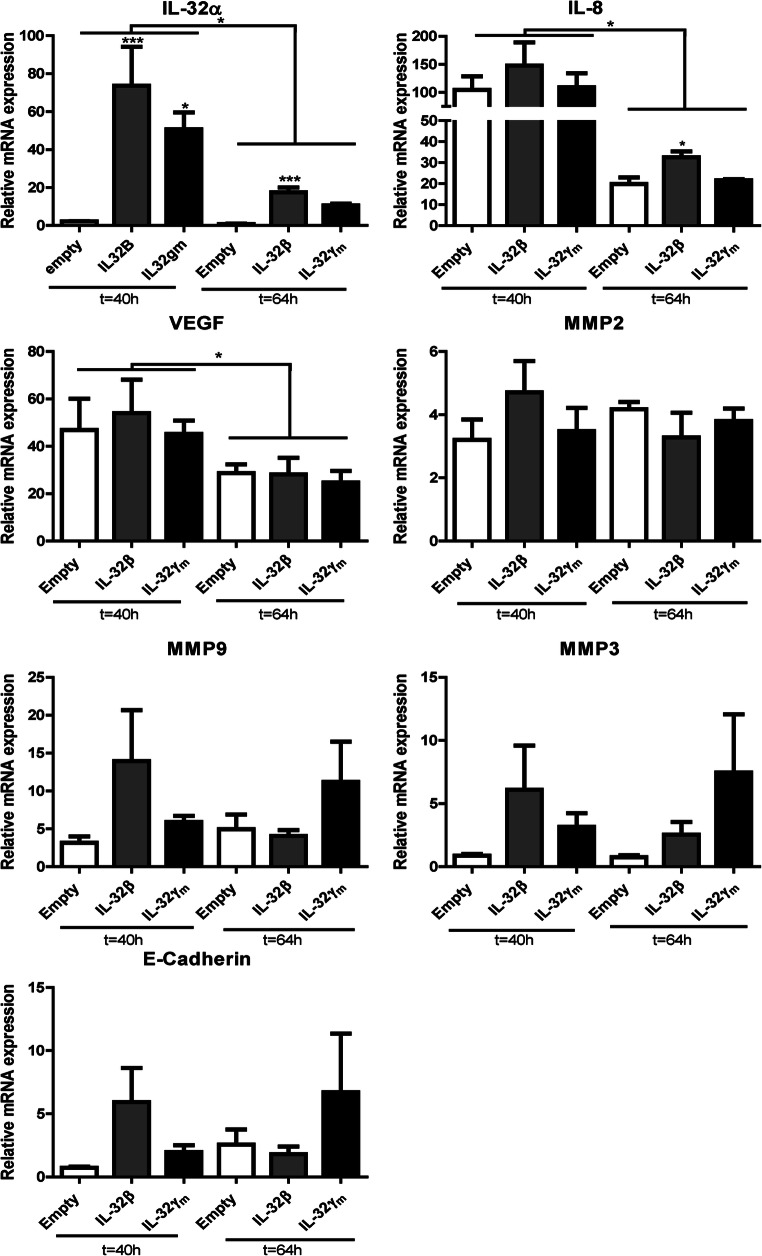
**mRNA expression of migratory factors associated with IL-32 induced cancer cell migration.** mRNA expression of IL-32α, IL-8, matrix metalloproteinases (MMP) 2, 3 and 9, epithelial-to-mesenchymal-transition (EMT) marker E-cadherin and vascular endothelial growth factor (VEGF) in the presence of 10% FCS. Results from 3 experiments, 2–3 replicates per experiment. Data are presented as mean ± SEM; * *p* < 0.05, ** *p* < 0.01, *** *p* < 0.001 by Mann-Whitney-U test or Kruskal Wallis test with Dunn’s multiple comparison test

Finally, we assessed the mRNA expression levels of factors known to be associated with IL-32 expression and cell migration in serum rich conditions. At 64 h after transfection, we found that the mRNA expression levels of IL-32α, IL-8 and VEGF were significantly reduced in all conditions compared to those 40 h after transfection (Fig. 5). The IL-32α and IL-8 mRNA expression levels were significantly upregulated in IL-32β-transfected cells at 64 h after transfection compared to those in control transfected cells (*p = 0.0003* and *p = 0.0120*). The IL-32α mRNA expression level was also significantly higher after 40 h in both IL-32β- and IL-32γ-transfected cells compared to control transfected cells. IL-32β and IL-32γ overexpression did not result in any significant differences in mRNA expression levels of MMP2, 3 or 9, E-cadherin or VEGF compared to control transfected cells at both time points.

## Discussion

In the present study, we show that IL-32 mRNA and protein expression in TC is strongly upregulated by TNFα derived from TC-induced macrophages, with IL-32β being the predominant isoform expressed by TC cells. Overexpression of IL-32β and IL-32γ in TC cells resulted in an increased cytotoxicity, without significantly affecting the expression of the proliferation marker Ki-67. Interestingly, we found that exogenous IL-32β overexpression in TC cells resulted in increased IL-8 mRNA expression, while other markers associated with cancer cell migration were not affected by either IL-32β or IL-32γ overexpression. Lastly, we found that exogenous IL-32β and IL-32γ overexpression did not affect TC cell migration in an in vitro model system.

An important finding was that IL-32 expression becomes highly upregulated in TC cells after co-culture with TLR-4-activated TC-induced macrophages. Especially the isoform IL-32β was found to be strongly induced after stimulation of TC cells with supernatants from activated TC-induced macrophages. Since TNFα is abundantly present in supernatants from activated TC-induced monocytes [21] and TNFα stimulation of TC cells results in the same pattern of induction of mainly IL-32β, it is reasonable to assume that TNFα is the main paracrine factor responsible for the induction of IL-32β in TC cells.

Interestingly, although TNFα has been shown to be a potent inducer of IL-32γ in human synovial fibroblasts [4], our results show that TNFα mainly induces IL-32β in TC cells and could, therefore, be considered an inducer of alternative splicing of IL-32 in TC. IL-32γ is considered to be the most potent isoform of IL-32 [3] and blocking of alternative splicing, resulting in predominantly IL-32γ expression, has been shown to induce cell death in FTC-133 and BCPAP TC cells [18]. In the present study, we have demonstrated that exogenous overexpression of IL-32γ significantly increased cytotoxicity in TPC-1 cells as well. These results indicate that IL-32γ may potently induce cell death in TC cells. Induction of alternative splicing by TNFα deduced from TC-induced macrophages, resulting in increased levels of IL-32β and IL-32α and reduced levels of IL-32γ, could protect TC cells from cell death. We found that IL-32β overexpression in TPC-1 cells resulted in slightly less cell death, and a significant increase in IL-8 mRNA expression, whereas IL-32γ did not. IL-8 is an important pro-survival cytokine [26] and restoring the IL-8 signalling pathway has been found to rescue IL-32β-induced cell death in HEK293 cells [18]. Our results further showed that the expression of Ki-67, an important marker of proliferation, was slightly reduced after exogenous IL-32γ overexpression, suggesting a reduced proliferation potential. These results support the hypothesis that stimulation of alternative splicing towards IL-32β may be beneficial for TC cells in terms of survival.

Exogenous IL-32β and IL-32γ overexpression in TPC-1 cells resulted in a high protein expression of the indicated isoforms. However, exogenous IL-32γ_mutant_ overexpression also resulted in IL-32β protein expression. This is surprising, since the IL-32γ_mutant_ pre-mRNA cannot be spliced. It is, therefore, likely that IL-32γ_mutant_ protein overexpression can induce the transcription of endogenous IL-32 mRNA in the transfected TCP-1 cells [2], which may result in increased levels of IL-32β mRNA as well, as evidenced by increased IL-32β protein expression at the later time point. In line with these findings, we observed lower levels of IL-32α mRNA in IL-32γ_mutant_ transfected cells compared to that in IL-32β transfected cells.

In contrast to what we hypothesized, we found that IL-32β and IL-32γ overexpression did not affect in vitro TC cell migration. Factors that have previously been found to be important in IL-32-induced cell migration [13–16], were also not significantly affected in TC cells after IL-32β and IL-32γ overexpression. Interestingly, however, we found that the mRNA expression levels of IL-8 and VEGF decreased over time, with significant lower expression levels 64 h after transfection. It is possible that the TPC-1 cells continued to proliferate, as indicated by Ki-67 expression, and became confluent after 64 h, resulting in contact inhibition, which could lead to entrance in a resting state and thus a reduced IL-8 and VEGF expression [27, 28].

In the present study, we only focused on the role of IL-32 in the interplay between TAMs and differentiated TC cells. However, TAMs are able to interact with other immune cells in the tumour micro-environment as well through release of soluble factors such as chemokines and cytokines (e.g. IL-6 and TNFα) [21]. The pro-inflammatory cytokines produced by TAMs in TC are known to stimulate adaptive immune responses. Interestingly, pro-inflammatory cytokines such as TNFα have also been shown to induce dedifferentiation of tumours, resulting in adaptive immune resistance [29, 30]. Thus, via an indirect route, TAMs may influence adaptive anti-tumour immune responses in TC as well. Interestingly, in the present study we found that TAM-derived TNFα induced IL-32 expression in TC cells and IL-32 expression in tumour cells is known to affect adaptive immune responses in several other solid cancers [9]. In vitro studies and animal models of different solid cancer types, including colon and prostate cancer, have revealed that IL-32β can stimulate the adaptive anti-tumour response by inducing NK cytotoxicity, thereby increasing T cell infiltration and stimulating a cytotoxic T cell response [12, 31, 32]. As this was beyond the scope of our study, future studies are warranted to assess the effect of IL-32 on adaptive anti-tumour responses in TC.

Although previous work has shown that TAM densities may be even higher in more advanced cancers, such as poorly differentiated TC and anaplastic TC [19, 20, 33], little experimental data are available on functional interactions between TAMs and TC cells in other subtypes of TC. For follicular TC, we have previously reported that co-culture with a follicular cancer cell line (FTC-133, PTEN-deficient) induced an even more proinflammatory phenotype in TC-induced macrophages compared to that by a papillary TC cell line (TPC-1, RET/ PTC rearrangement) [34]. These results indicate that IL-32β may be more strongly induced in follicular TC, as the levels of TAM-derived TNFα are also significantly higher and, thus, could play a more important role in follicular TC. However, future studies are needed to investigate the functional interactions between TAMs and other subtypes of TC, as well as the effect of IL-32 in other subtypes of TC.

In conclusion, we found that TAM-derived TNFα is the main inducer of IL-32 alternative splicing in TC cells, resulting in upregulation of IL-32β expression. Although IL-32β does not affect TC cell migration, alternative splicing of IL-32 towards IL-32β is likely beneficial for TC cell survival by reducing IL-32γ-induced cell death and inducing increased levels of the pro-survival cytokine IL-8. Therefore, modulation of IL-32 alternative splicing could be explored as potential novel treatment strategy for patients with advanced TC.

## Electronic supplementary material

ESM 1 (PDF 567 kb)

## Abbreviations

EMT: Epithelial-to-mesenchymal-transition
GFP: Green fluorescent protein
IL-32: Interleukin 32
MMP: Matrix metalloproteinase
PBMCs: Peripheral blood mononuclear cells
SNP: Single nucleotide polymorphism
TAMs: Tumour associated macrophages
TC: Non-medullary thyroid cancer
TME: Tumour microenvironment
TNFα: Tumour necrosis factor α
VEGF: Vascular endothelial growth factor

## References

[1] S.H. Kim, S.Y. Han, T. Azam, D.Y. Yoon, C.A. Dinarello, Interleukin-32: A cytokine and inducer of TNFalpha. Immunity **22**, 131–142 (2005). 10.1016/j.immuni.2004.12.003 15664165 10.1016/j.immuni.2004.12.003

[2] B. Heinhuis, M.I. Koenders, F.A. van de Loo, M.G. Netea, W.B. van den Berg, L.A. Joosten, Inflammation-dependent secretion and splicing of IL-32{gamma} in rheumatoid arthritis. Proc Natl Acad Sci U S A **108**, 4962–4967 (2011). 10.1073/pnas.1016005108 21383200 10.1073/pnas.1016005108 PMC3064318

[3] L.A. Joosten, B. Heinhuis, M.G. Netea, C.A. Dinarello, Novel insights into the biology of interleukin-32. Cell Mol Life Sci **70**, 3883–3892 (2013). 10.1007/s00018-013-1301-9 23463238 10.1007/s00018-013-1301-9 PMC11113358

[4] B. Heinhuis, M.I. Koenders, P.L. van Riel, F.A. van de Loo, C.A. Dinarello, M.G. Netea, W.B. van den Berg, L.A. Joosten, Tumour necrosis factor alpha-driven IL-32 expression in rheumatoid arthritis synovial tissue amplifies an inflammatory cascade. Ann Rheum Dis **70**, 660–667 (2011). 10.1136/ard.2010.139196 21187297 10.1136/ard.2010.139196

[5] B. Heinhuis, C.D. Popa, B.L. van Tits, S.H. Kim, P.L. Zeeuwen, W.B. van den Berg, J.W. van der Meer, J.A. van der Vliet, A.F. Stalenhoef, C.A. Dinarello, M.G. Netea, L.A. Joosten, Towards a role of interleukin-32 in atherosclerosis. Cytokine **64**, 433–440 (2013). 10.1016/j.cyto.2013.05.002 23727326 10.1016/j.cyto.2013.05.002

[6] J.T. Hong, D.J. Son, C.K. Lee, D.Y. Yoon, D.H. Lee, M.H. Park, Interleukin 32, inflammation and cancer. Pharmacol Ther **174**, 127–137 (2017). 10.1016/j.pharmthera.2017.02.025 28223235 10.1016/j.pharmthera.2017.02.025

[7] A.M. Marcondes, A.J. Mhyre, D.L. Stirewalt, S.H. Kim, C.A. Dinarello, H.J. Deeg, Dysregulation of IL-32 in myelodysplastic syndrome and chronic myelomonocytic leukemia modulates apoptosis and impairs NK function. Proc Natl Acad Sci U S A **105**, 2865–2870 (2008). 10.1073/pnas.0712391105 18287021 10.1073/pnas.0712391105 PMC2268551

[8] C. Sorrentino, E. Di Carlo, Expression of IL-32 in human lung cancer is related to the histotype and metastatic phenotype. Am J Respir Crit Care Med **180**, 769–779 (2009). 10.1164/rccm.200903-0400OC 19628777 10.1164/rccm.200903-0400OC

[9] Y.J.E. Sloot, J.W. Smit, L.A.B. Joosten, R.T. Netea-Maier, Insights into the role of IL-32 in cancer. Semin Immunol **38**, 24–32 (2018). 10.1016/j.smim.2018.03.004 29747940 10.1016/j.smim.2018.03.004

[10] J.H. Oh, M.C. Cho, J.H. Kim, S.Y. Lee, H.J. Kim, E.S. Park, J.O. Ban, J.W. Kang, D.H. Lee, J.H. Shim, S.B. Han, D.C. Moon, Y.H. Park, D.Y. Yu, J.M. Kim, S.H. Kim, D.Y. Yoon, J.T. Hong, IL-32gamma inhibits cancer cell growth through inactivation of NF-kappaB and STAT3 signals. Oncogene **30**, 3345–3359 (2011). 10.1038/onc.2011.52 21423208 10.1038/onc.2011.52 PMC3145890

[11] E.S. Park, J.M. Yoo, H.S. Yoo, D.Y. Yoon, Y.P. Yun, J. Hong, IL-32gamma enhances TNF-alpha-induced cell death in colon cancer. Mol Carcinog **53**(Suppl 1), E23–E35 (2014). 10.1002/mc.21990 23255489 10.1002/mc.21990

[12] H.M. Yun, J.H. Oh, J.H. Shim, J.O. Ban, K.R. Park, J.H. Kim, D.H. Lee, J.W. Kang, Y.H. Park, D. Yu, Y. Kim, S.B. Han, D.Y. Yoon, J.T. Hong, Antitumor activity of IL-32beta through the activation of lymphocytes, and the inactivation of NF-kappaB and STAT3 signals. Cell Death Dis **4**, e640 (2013). 10.1038/cddis.2013.166 23703385 10.1038/cddis.2013.166 PMC3674373

[13] J.S. Park, S.Y. Choi, J.H. Lee, M. Lee, E.S. Nam, A.L. Jeong, S. Lee, S. Han, M.S. Lee, J.S. Lim, D.Y. Yoon, Y. Kwon, Y. Yang, Interleukin-32beta stimulates migration of MDA-MB-231 and MCF-7cells via the VEGF-STAT3 signaling pathway. Cell Oncol **36**, 493–503 (2013). 10.1007/s13402-013-0154-4 10.1007/s13402-013-0154-4 PMC13007480 24114327

[14] C.Y. Tsai, C.S. Wang, M.M. Tsai, H.C. Chi, W.L. Cheng, Y.H. Tseng, C.Y. Chen, C.D. Lin, J.I. Wu, L.H. Wang, K.H. Lin, Interleukin-32 increases human gastric cancer cell invasion associated with tumor progression and metastasis. Clin Cancer Res **20**, 2276–2288 (2014). 10.1158/1078-0432.ccr-13-1221 24602839 10.1158/1078-0432.CCR-13-1221

[15] Q. Zeng, S. Li, Y. Zhou, W. Ou, X. Cai, L. Zhang, W. Huang, L. Huang, Q. Wang, Interleukin-32 contributes to invasion and metastasis of primary lung adenocarcinoma via NF-kappaB induced matrix metalloproteinases 2 and 9 expression. Cytokine **65**, 24–32 (2014). 10.1016/j.cyto.2013.09.017 24140068 10.1016/j.cyto.2013.09.017

[16] Y. Zhou, Z. Hu, N. Li, R. Jiang, Interleukin-32 stimulates osteosarcoma cell invasion and motility via AKT pathway-mediated MMP-13 expression. Int J Mol Med **35**, 1729–1733 (2015). 10.3892/ijmm.2015.2159 25846944 10.3892/ijmm.2015.2159

[17] T.S. Plantinga, I. Costantini, B. Heinhuis, A. Huijbers, G. Semango, B. Kusters, M.G. Netea, A.R. Hermus, J.W. Smit, C.A. Dinarello, L.A. Joosten, R.T. Netea-Maier, A promoter polymorphism in human interleukin-32 modulates its expression and influences the risk and the outcome of epithelial cell-derived thyroid carcinoma. Carcinogenesis **34**, 1529–1535 (2013). 10.1093/carcin/bgt092 23486016 10.1093/carcin/bgt092

[18] B. Heinhuis, T.S. Plantinga, G. Semango, B. Kusters, M.G. Netea, C.A. Dinarello, J.W.A. Smit, R.T. Netea-Maier, L.A.B. Joosten, Alternatively spliced isoforms of IL-32 differentially influence cell death pathways in cancer cell lines. Carcinogenesis **37**, 197–205 (2016). 10.1093/carcin/bgv172 26678222 10.1093/carcin/bgv172

[19] B. Caillou, M. Talbot, U. Weyemi, C. Pioche-Durieu, A. Al Ghuzlan, J.M. Bidart, S. Chouaib, M. Schlumberger, C. Dupuy, Tumor-associated macrophages (TAMs) form an interconnected cellular supportive network in anaplastic thyroid carcinoma. PLoS One **6**, e22567 (2011). 10.1371/journal.pone.0022567 21811634 10.1371/journal.pone.0022567 PMC3141071

[20] M. Ryder, R.A. Ghossein, J.C. Ricarte-Filho, J.A. Knauf, J.A. Fagin, Increased density of tumor-associated macrophages is associated with decreased survival in advanced thyroid cancer. Endocr Relat Cancer **15**, 1069–1074 (2008). 10.1677/erc-08-0036 18719091 10.1677/ERC-08-0036 PMC2648614

[21] R.J.W. Arts, T.S. Plantinga, S. Tuit, T. Ulas, B. Heinhuis, M. Tesselaar, Y. Sloot, G.J. Adema, L.A.B. Joosten, J.W.A. Smit, M.G. Netea, J.L. Schultze, R.T. Netea-Maier, Transcriptional and metabolic reprogramming induce an inflammatory phenotype in non-medullary thyroid carcinoma-induced macrophages. Oncoimmunology **5**, e1229725 (2016). 10.1080/2162402X.2016.1229725 10.1080/2162402X.2016.1229725 PMC5213309 28123869

[22] R.E. Schweppe, J.P. Klopper, C. Korch, U. Pugazhenthi, M. Benezra, J.A. Knauf, J.A. Fagin, L.A. Marlow, J.A. Copland, R.C. Smallridge, B.R. Haugen, Deoxyribonucleic acid profiling analysis of 40 human thyroid cancer cell lines reveals cross-contamination resulting in cell line redundancy and misidentification. J Clin Endocrinol Metab **93**, 4331–4341 (2008). 10.1210/jc.2008-1102 18713817 10.1210/jc.2008-1102 PMC2582569

[23] J.T. Leek, W.E. Johnson, H.S. Parker, A.E. Jaffe, J.D. Storey, The sva package for removing batch effects and other unwanted variation in high-throughput experiments. Bioinformatics **28**, 882–883 (2012). 10.1093/bioinformatics/bts034 22257669 10.1093/bioinformatics/bts034 PMC3307112

[24] M.E. Ritchie, B. Phipson, D. Wu, Y. Hu, C.W. Law, W. Shi, G.K. Smyth, Limma powers differential expression analyses for RNA-sequencing and microarray studies. Nucleic Acids Res **43**, e47 (2015). 10.1093/nar/gkv007 25605792 10.1093/nar/gkv007 PMC4402510

[25] J.T. Robinson, H. Thorvaldsdóttir, W. Winckler, M. Guttman, E.S. Lander, G. Getz, J.P. Mesirov, Integrative genomics viewer. Nat Biotechnol **29**, 24 (2011). 10.1038/nbt.1754https://www.nature.com/articles/nbt.1754#supplementary-information 21221095 10.1038/nbt.1754 PMC3346182

[26] M. Rotondi, F. Coperchini, F. Latrofa, L. Chiovato, Role of chemokines in thyroid Cancer microenvironment: Is CXCL8 the Main player? Front Endocrinol (Lausanne) **9**(314) (2018). 10.3389/fendo.2018.00314 10.3389/fendo.2018.00314 PMC6021500 29977225

[27] F. Fagotto, B.M. Gumbiner, Cell contact-dependent signaling. Dev Biol **180**, 445–454 (1996). 10.1006/dbio.1996.0318 8954717 10.1006/dbio.1996.0318

[28] F. Vinals, J. Pouyssegur, Confluence of vascular endothelial cells induces cell cycle exit by inhibiting p42/p44 mitogen-activated protein kinase activity. Mol Cell Biol **19**, 2763–2772 (1999) 10082542 10.1128/mcb.19.4.2763 PMC84069

[29] J. Landsberg, J. Kohlmeyer, M. Renn, T. Bald, M. Rogava, M. Cron, M. Fatho, V. Lennerz, T. Wolfel, M. Holzel, T. Tuting, Melanomas resist T-cell therapy through inflammation-induced reversible dedifferentiation. Nature **490**, 412–416 (2012). 10.1038/nature11538 23051752 10.1038/nature11538

[30] A. Mehta, Y.J. Kim, L. Robert, J. Tsoi, B. Comin-Anduix, B. Berent-Maoz, A.J. Cochran, J.S. Economou, P.C. Tumeh, C. Puig-Saus, A. Ribas, Immunotherapy resistance by inflammation-induced dedifferentiation. Cancer Discov **8**, 935–943 (2018). 10.1158/2159-8290.Cd-17-1178 29899062 10.1158/2159-8290.CD-17-1178 PMC6076867

[31] S. Bhat, N. Gardi, S. Hake, N. Kotian, S. Sawant, S. Kannan, V. Parmar, S. Desai, A. Dutt, N.N. Joshi, Impact of intra-tumoral IL17A and IL32 gene expression on T-cell responses and lymph node status in breast cancer patients. J Cancer Res Clin Oncol **143**, 1745–1756 (2017). 10.1007/s00432-017-2431-5 28470472 10.1007/s00432-017-2431-5 PMC5863950

[32] M.H. Park, M.J. Song, M.C. Cho, D.C. Moon, D.Y. Yoon, S.B. Han, J.T. Hong, Interleukin-32 enhances cytotoxic effect of natural killer cells to cancer cells via activation of death receptor 3. Immunology **135**, 63–72 (2012). 10.1111/j.1365-2567.2011.03513.x 22043900 10.1111/j.1365-2567.2011.03513.x PMC3246653

[33] K.Y. Jung, S.W. Cho, Y.A. Kim, D. Kim, B.C. Oh, D.J. Park, Y.J. Park, Cancers with higher density of tumor-associated macrophages were associated with poor survival rates. J Pathol Transl Med **49**, 318–324 (2015). 10.4132/jptm.2015.06.01 26081823 10.4132/jptm.2015.06.01 PMC4508569

[34] Y.J.E. Sloot, K. Rabold, M.G. Netea, J.W.A. Smit, N. Hoogerbrugge, R.T. Netea-Maier, Effect of PTEN inactivating germline mutations on innate immune cell function and thyroid cancer-induced macrophages in patients with PTEN hamartoma tumor syndrome. Oncogene (2019). 10.1038/s41388-019-0685-x 10.1038/s41388-019-0685-x 30670777

