# Supplementary material for: Interplay between thyroid cancer cells and macrophages: effects on IL-32 mediated cell death and thyroid cancer cell migration
Source: Cell Oncol (Dordr). 2019 Jun 14;42(5):691–703. doi: 10.1007/s13402-019-00457-9 (PMC12994284; doi:10.1007/s13402-019-00457-9)
Supplement: Supplementary file 1 — (PDF 567 kb) [file 13402_2019_457_MOESM1_ESM.pdf]

SUPPLEMENT

Interplay between thyroid cancer cells and macrophages: effects on IL-32 mediated cell death and thyroid cancer cell migration.

Yvette J.E. Sloot, MD, Katrin Rabold, MSc, Thomas Ulas PhD, Dennis M. De Graaf MSc, Bas Heinhuis, PhD, Kristian Händler, PhD, Joachim L. Schultze, MD, PhD, Mihai G. Netea, MD, PhD., Johannes W.A. Smit, MD PhD, Leo A.B. Joosten, PhD, Romana T. Netea-Maier, MD, PhD.

Supplementary Figures

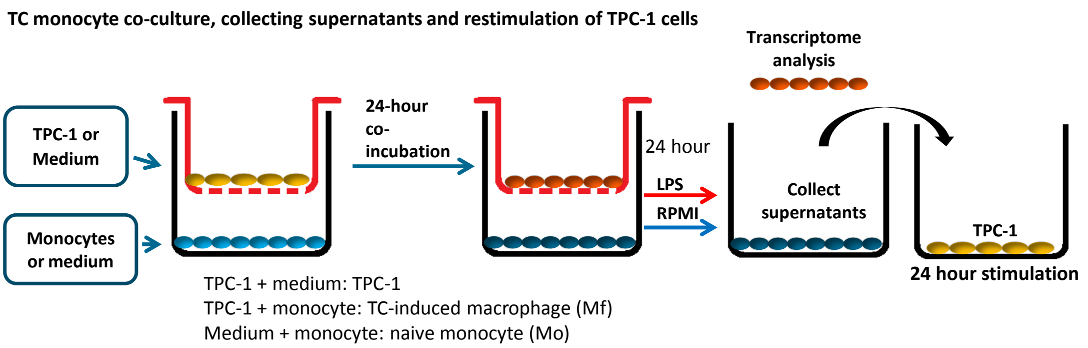

Supplementary Figure S1 Experimental Set-up. The upper well contains TPC-1 cells or Medium, while the bottom well contains monocytes or medium. TC cells and monocytes are co-cultured for 24 hours, creating naive TPC-1 cells, TC-induced macrophages (Mf) and Naive monoctes (mo). Next, LPS (TLR-4 ligand) or RPMI (medium control) were added to the bottom well for 24 hours. TPC-1 cells are collected for transcriptome analysis, supernatants are collected for conditioned medium experiments.

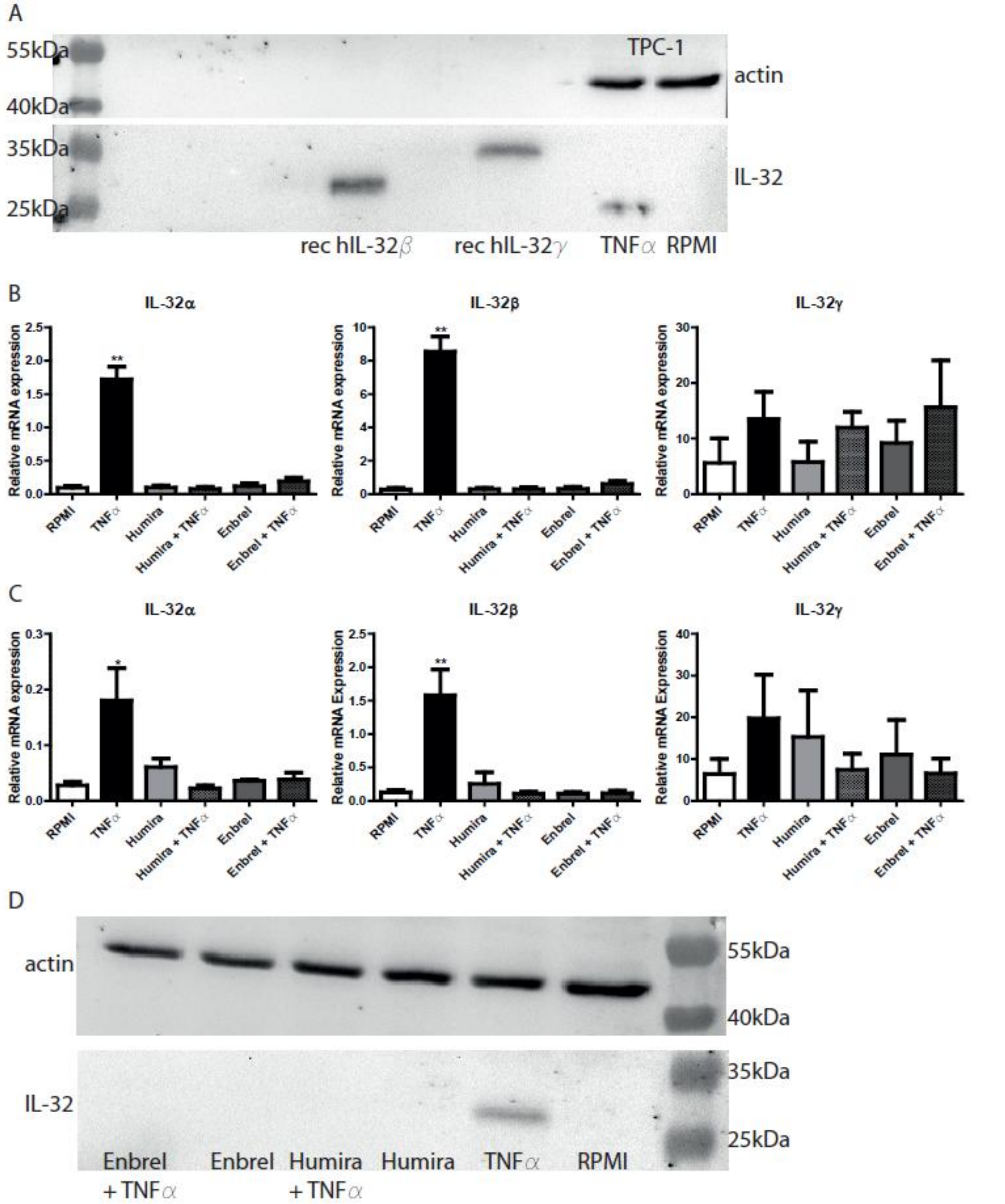

Supplementary Figure S2 TNFα-mediated IL-32 mRNA and protein expression in different TC cell lines. A) Western blot analysis demonstrating IL-32β protein expression in TPC-1 cells; Human recombinant IL-32β and IL-32γ was loaded on the blot as control. B) Relative mRNA expression of IL-32α, IL-32β and IL-32γ isoforms in FTC-133 cells after stimulation with TNFα and in the presence of Enbrel (etanercept, decoy TNFα-receptor, 10 ug/ml) or Humira

(adalimumab, monoclonal TNFα antibody, 10 ug/ml). C) Relative mRNA expression of IL-32α, IL-32β and IL-32γ isoforms in BC-PAP cells after stimulation with TNFα and in the presence of Enbrel or Humira. Results from 3 experiments, 1-3 replicates per experiment. E) Western blot analysis showing IL-32 protein expression in FTC-133 after stimulation with TNFα and in the presence of Enbrel or Humira. Data are represented as mean ± SEM; * $p<0.05$, ** $p<0.01$, by Mann-Whitney-U test or Kruskal-Wallis test with Dunn's multiple comparison test.

Supplemental methods

Western blot using human recombinant IL-32β and IL-32γ

human recombinant IL-32β and IL-32γ (R&D systems, Minneapolis, MN, USA) was diluted to a concentration of 2,5 ng/ml. A total of 100 pg/lane was loaded on a pre-casted 4-15% gel (Bio-Rad, CA, USA) for polyacrylamide gel electrophoresis. Further procedure described in manuscript.

Supplementary table S1 Primer sequences

Target	Forward 5’> 3’	Reverse 5’> 3’
IL-32α	GCTGGAGGACGACTTCAAAGA	GGGCTCCGTAGGACTTGTCA
MMP2	TACAGGATCATTGGCTACACACC	GGTCACATCGCTCCAGACT
MMP3	CTGGACTCCGACACTCTGGA	CAGGAAAGGTTCTGAAGTGACC
MMP9	TGTACCGCTATGGTTACACTCG	GGCAGGGACAGTTGCTTCT
IL-8	ACTGAGAGTGATTGAGAGTGGAC	AACCCTCTGCACCCAGTTTTC
E-cadherin	AAAGGCCCATTTCCTAAAAACCT	TGCGTTCTCTATCCAGAGGCT
VEGF	AGGGCAGAATCATCACGAAGT	AGGGTCTCGATTGGATGGCA
B2M	ATGAGTATGCCTGCCGTGTG	CCAAATGCGGCATCTTCAAAC
GAPDH	AGGGAGATTCAGTGTGGTG	CGACCACTTTGTCAAGCTCA
